# Definitive Proton Beam Therapy for Second Primary Non–small Cell Lung Cancer After Pneumonectomy

**DOI:** 10.1016/j.ijpt.2025.101202

**Published:** 2025-09-15

**Authors:** Masatoshi Nakamura, Kayoko Ohnishi, Toshiyuki Okumura, Den Fujioka, Keiichiro Baba, Motohiro Murakami, Masashi Mizumoto, Ikuo Sekine, Masaharu Inagaki, Yukio Sato, Hideyuki Sakurai

**Affiliations:** a Department of Radiation Oncology, Institute of Medicine, University of Tsukuba, Tsukuba, Japan; b Department of Radiology, School of Medicine, International University of Health and Welfare, Narita, Japan; c Department of Radiation Oncology, Ibaraki Prefectural Central Hospital, Kasama, Japan; d Department of Medical Oncology, Institute of Medicine, University of Tsukuba, Tsukuba, Japan; e Department of Thoracic Surgery, Tsuchiura Kyodo General Hospital, Tsuchiura, Japan; f Department of Thoracic Surgery, Institute of Medicine, University of Tsukuba, Tsukuba, Japan

**Keywords:** Proton beam therapy, Pneumonectomy, Non–small cell lung cancer, Dose-volume histogram

## Abstract

**Purpose:**

To investigate the outcome of definitive proton beam therapy (PBT) for second primary non–small cell lung cancer after pneumonectomy.

**Patients and Methods:**

Between January 2006 and December 2019, 4 patients with second primary lung cancer occurring in the contralateral lung after pneumonectomy were selected from our database of patients who underwent PBT. All patients were men with a median age of 69 (range, 61-79) years at the time of PBT. The median time from pneumonectomy to PBT initiation was 104.5 (range, 92-117) months. All patients had a histology of squamous cell carcinoma and were inoperable due to poor respiratory function.

**Results:**

The median survival time was 46.6 (range, 11.7-118.4) months; 3 patients died of their primary disease and one of bacterial pneumonia. The first relapses were local recurrence inside the irradiated field (*n* = 1) and outside the irradiated field (*n* = 2) and regional (*n* = 1). The median time to relapse was 21.5 (range, 6.6-48.1) months. Regarding adverse events, grade 2 pneumonitis occurred in 2 patients, but steroid administration was not required; no grade ≥3 pneumonitis was observed.

**Conclusion:**

Definitive PBT for new lesions in the residual lung after pneumonectomy was successful without serious adverse events, although the number of cases was small.

## Introduction

The incidence of intrapulmonary relapse after radical surgery for non–small cell lung cancer is reported to be 6% to 55%, and that of metachronous primary lung cancer is approximately 10%.^1^, ^2^, ^3^ Because of the large loss of respiratory function and the high risks of postoperative complications and death, pneumonectomy is generally avoided by sleeve resection if possible, and consequently, there are fewer cases of pneumonectomy these days.^4^, ^5^ However, there have been cases of new lung lesions occurring in the remaining lung nearly 10 years after surgery, as well as cases of lung cancer developing after pneumonectomy for other lung diseases.^1^, ^2^, ^3^, ^4^, ^5^ Therefore, it is sometimes necessary to treat lung cancer cases after pneumonectomy.

Radiation therapy is considered the definitive treatment for patients who develop lung cancer after pneumonectomy, as surgery is often difficult due to postoperative complications or pulmonary function issues.^6^ However, there are few reports on the efficacy and safety of radiation therapy for these patients.^7^

Proton beam therapy (PBT) provides better dose distribution than does x-ray radiation therapy owing to the sharp dose fall-off beyond the Bragg peak.^8^ Thus, PBT is theoretically safer than x-ray radiation therapy for lung cancer patients after pneumonectomy.^9^ Here, we describe our experience to determine the potential safety and effectiveness of PBT as a curative treatment for metachronous primary lung cancer in the residual lung after pneumonectomy.

## Patients and methods

### Patients

Between January 2006 and December 2019, 4 patients with primary lung cancer occurring in the contralateral lung after pneumonectomy received definitive PBT at our institution. We used the criteria proposed by Martini et al^1^ to evaluate whether the newly developed lung nodules were primary lung cancer or metastasis. The patient characteristics are shown in Table 1. All 4 patients were men with a median age of 59.5 (range, 52-71) years at the time of pneumonectomy. The pathological histology was squamous cell carcinoma (SCC) in 3 patients and large cell carcinoma in one. The pathological stage, according to the 8th edition of the Union for International Cancer Control TNM classification, was IIB in 2 patients, IIIA in 1 patient, and IIIB in 1 patient. Two patients received perioperative radiation therapy. The median time from pneumonectomy to the start of PBT was 104.5 (range, 92-117) months, and the median age was 69 (range, 61-79) years at the time of PBT. Of the 4 patients, one had an Eastern Cooperative Oncology Group performance status (ECOG PS) of 0, and 3 had an ECOG PS of 1; all were ambulatory. None of the patients were receiving home oxygen therapy prior to PBT. Second primary histology was SCC in all 4 patients, and the clinical stages were IA in 1 patient, IIA in 2 patients, and IIIA in 1 patient. All 4 patients were inoperable due to poor respiratory function; the vital capacity ranged from 1.51 to 1.76 L, and the forced expiratory volume in 1 s ranged from 0.87 to 1.18 L.

**Table 1:** Patient, tumor, and treatment characteristics.

Case	Age at surgery (year)	Primary site at surgery	Histology at surgery	Pathological stage (UICC 8th)	Age at PBT (year)	ECOG PS at PBT	VC before PBT (L)	FEV1.0 before PBT (L)	Primary site at PBT	Histology at PBT	Clinical stage (UICC 8th)	Prescribed dose
1	52	Left S1+2	LC	pT4N2M0	61	0	1.76	1.11	S7	SCC	cT4N1M0	70.0 Gy (RBE) in 35 Fx
2	61	Left hilum	SCC	ypT2aN1M0	71	1	1.72	1.18	S2	SCC	cT1cN0M0	72.6 Gy (RBE) in 22 Fx
3	58	Left S4/5, S8	SCC	pT2bN1, pT1b	67	1	1.78	0.87	S8	SCC	cT2bN0M0	72.6 Gy (RBE) in 22 Fx
4	71	Left S6	SCC	ypT4N1M0	79	1	1.51	1.03	S3	SCC	cT2bN0M0	66.0 Gy (RBE) in 10 Fx

**Abbreviations**: ECOG, Eastern Cooperative Oncology Group; FEV1.0, forced expiratory volume in 1 s; Fx, fractions; LC, large cell carcinoma; PBT, proton beam therapy; PS, performance status; RBE, relative biological effectiveness; SCC, squamous cell carcinoma; UICC, Union for International Cancer Control; VC, vital capacity.

### Proton beam therapy

Passive-scattering PBT was delivered during the end-expiratory phase via a respiratory-gated system using 155 to 250 MeV protons. The patient's body was immobilized using a custom-shaped body cast (ESFORM, Engineering System Co). Prior to each treatment, the patient's position was confirmed by fluoroscopy. For treatment planning, chest computed tomography (CT) images were obtained in 2.5- or 5-mm slice thickness in the treatment position using a respiratory-gated system during the end-expiratory phase. The clinical target volume encompassed only the gross tumor for all 4 patients, and the planning target volume encompassed the clinical target volume plus 7- to 10-mm margins in all directions plus an additional 5-mm margin in the caudal direction to compensate for respiratory motion. The dose calculation was performed using the pencil beam method for PBT (proton treatment planning software ver. 2, Hitachi Inc). The prescribed doses were 66.0 Gy (relative biological effectiveness [RBE]) in 10 fractions for peripherally located stage IIA disease in 1 patient, 72.6 Gy (RBE) in 22 fractions for centrally located stage IA or IIA disease in 2 patients, and 70.0 Gy (RBE) in 35 fractions for stage IIIA disease in 1 patient. No patient received chemotherapy or immunotherapy before or after PBT.

### Follow-up

Post-treatment evaluation was performed every 1 to 2 months during the first year and every 3 to 4 months thereafter. The follow-up examinations included physical examinations, blood tests, chest x-rays, and CT or PET/CT scans. The follow-up interval was defined from the first day of PBT to the date of the event or last follow-up. Adverse events (AEs) were assessed according to the Common Terminology Criteria for Adverse Events version 5.0.^10^

### Comparison of dose-volume histograms of the lung between proton and x-ray radiation therapy

We created a virtual x-ray treatment plan based on the same treatment planning CT used at the time of PBT for each patient, to compare lung doses between proton and x-ray radiation therapy. The dose calculation was performed using collapsed cone convolution for x-ray radiation therapy (Raystation v10.0.1, RaySearch Laboratories). We developed a treatment plan for intensity modulated radiation therapy using x-rays in 1 patient with stage IIIA disease and a stereotactic body radiation therapy (SBRT) plan with 7 to 8 fixed multiportals in 3 patients with stage IA or IIA disease.

## Results

### Treatment efficacy and adverse events

A summary of the outcomes and AEs after PBT is shown in Table 2. All 4 patients died, with a median survival time of 46.6 (range, 11.7-118.4) months; 3 patients died of their primary disease and 1 patient of bacterial pneumonia. The first relapse sites included local recurrence inside the irradiated field in 1 patient, local recurrence outside the irradiated field in 2 patients, and regional recurrence in 1 patient, with a median time to relapse of 21.5 (range, 6.6-48.1) months. The median progression-free survival time was 21.5 (range, 6.6-48.1) months, and the time to local recurrence inside the irradiated field in the single affected patient was 48.1 months.

**Table 2:** Outcomes and adverse events after proton beam therapy.

Case	Time to initial recurrence after PBT (months)	Recurrence pattern	Retreatment	Number of reirradiations with PBT	Survival (months)	Cause of death	Pneumonitis grade	VC after PBT (L)	FEV1.0 after PBT (L)	ECOG PS 1 y after PBT	HOT after PBT
1	6.6	Local (out-field)	Electrocauterization Reirradiation	1	118.4	Bacterial pneumonia	2	1.62	1.06	0	No
2	11.6	Regional LN (out-field)	BSC	0	11.7	Cancer-related	1	-	-	-	No
3	48.1	Local (in-field)	BSC	0	50.3	Cancer-related	1	1.08	0.52	1	Yes
4	31.4	Local (out-field)	Reirradiation	2	42.9	Cancer-related	2	1.16	0.86	1	No

**Abbreviations**: ECOG, Eastern Cooperative Oncology Group; BSC, best supportive care; FEV1.0, forced expiratory volume in 1 s; HOT, home oxygen therapy; LN, lymph node; PBT, proton beam therapy; PS, performance status; VC, vital capacity.

Regarding AEs, no grade 3 or severe pneumonitis was observed. Grade 2 pneumonitis occurred in 2 patients, but administration of steroids was not required. In 3 of the 4 patients who survived at least 1 year, respiratory function tests were comparable before PBT and over 1 year after PBT: The median decrease in the vital capacity was 0.35 (range, 0.14-0.70) L, and that in the forced expiratory volume in 1 s was 0.17 (range, 0.05-0.35) L. Home oxygen therapy was initiated 6 months after PBT in one of the 3 surviving patients because of influenza virus infection; however, none of the 3 patients experienced a decline in ECOG PS 1 year after PBT.

### Representative case presentation: Case 1

**Figure 1:**
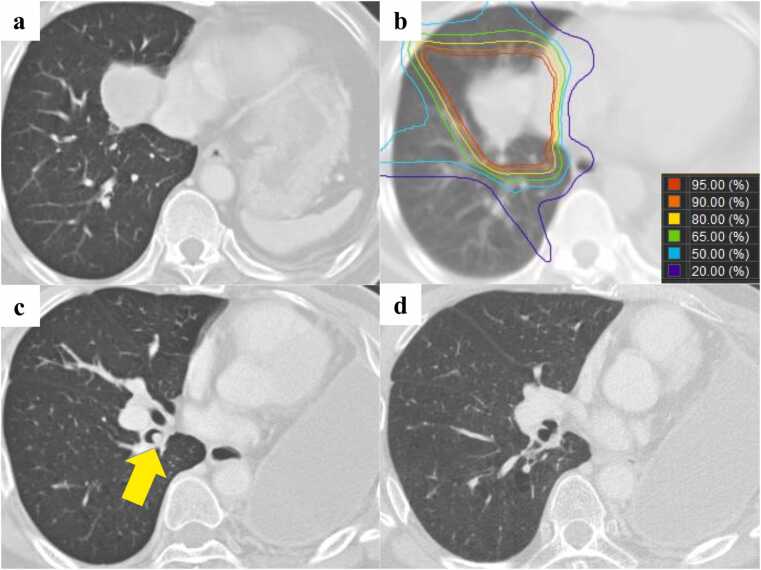
Computed tomography images of case 1 at the time of the first proton beam therapy (PBT) and at recurrence after PBT. a) The primary tumor invading the mediastinum in the right S7 before the initial PBT. b) The dose distribution of PBT. c) Recurrence in the mucosa of the right B7 at 7 months after PBT (arrow). d) The right B7 after bronchoscopic electrocautery for the recurrence.

**Figure 2:**
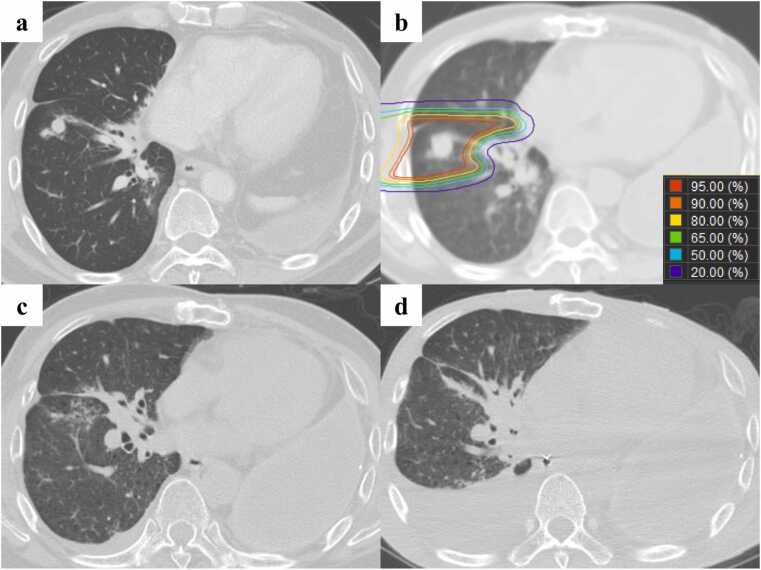
Computed tomography images of case 1 at the time of and after the second proton beam therapy round. a) A newly developed squamous cell carcinoma in the right S9 at 53 months after the initial PBT. b) The dose distribution of the second round of PBT delivered to the right S9 tumor. c) Radiation fibrosis without local progression in the irradiated field at 43 months after the second PBT round. d) Bacterial pneumonia in the right lower lobe, which was the cause of death, at 60 months after PBT.

A 52-year-old man received pneumonectomy for large cell carcinoma in the left upper lobe. The pathological stage was T4N2M0 stage IIIB, and postoperative radiation therapy consisted of 60 Gy in 30 fractions using x-rays due to a positive resection margin on the pericardial dissected surface. Nine years later, the patient developed SCC in the right S7 with clinical stage T4N1M0 stage IIIA (Figure 1). He received PBT of 70 Gy (RBE) in 35 fractions without concurrent chemotherapy. Grade 2 pneumonitis occurred 4 months after PBT, but administration of steroids was not required. At 7 months after PBT, recurrence occurred in the mucosa of the right B7 within the irradiated area, and bronchoscopic electrocautery was performed; the patient was then followed up without further treatment. At 53 months after PBT, he was diagnosed with newly developed SCC of clinical stage T1bN0M0 in the right S9, and he received hypofractionated PBT consisting of 72.6 Gy (RBE) in 22 fractions (Figure 2). The pneumonitis became grade 1 after the second PBT round. There was no recurrence thereafter, but he died of bacterial pneumonia at 118 months after the initial PBT.

### Representative case presentation: Case 2

**Figure 3:**
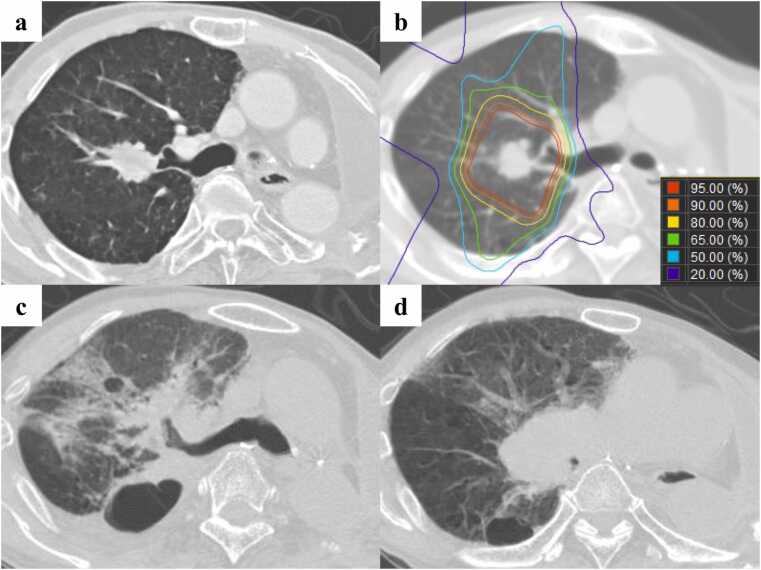
Computed tomography images of case 2. a) A primary tumor in the right S2 before proton beam therapy (PBT) b) The dose distribution of PBT. c) Radiation fibrosis without progression of the primary tumor at 12 months after PBT. d) Stenosis of the bronchus intermedius due to the right hilar lymph node recurrence at 12 months after PBT.

A 61-year-old man received pneumonectomy for SCC in the left hilum. Neoadjuvant chemoradiotherapy, which included cisplatin (50 mg/m^2^) and etoposide (50 mg/m^2^) combined with x-rays (45 Gy in 25 fractions), was performed due to suspected infiltration of the inferior pulmonary vein. The pathological stage was ypT2aN1M0 stage IIB. Ten years later, the patient developed SCC in the right S2 of clinical stage T1cN0M0 stage IA3 (Figure 3). He received hypofractionated PBT of 72.6 Gy (RBE) in 22 fractions. Twelve months after PBT, he developed asthma-like dyspnea, and CT revealed stenosis of the bronchus intermedius, right middle lobe bronchus, and right lower lobe bronchus due to the right hilar lymph node recurrence, which was outside the irradiated field. Two days later, he died of obstructive pneumonia caused by the recurrences.

### Comparison of treatment plans between proton and x-rays for lung dose and heart dose

The dosimetric parameters of the lung and heart comparing the PBT and virtual x-ray plans are shown in Table 3. The median lung V5 and V20, defined as the percentages of the lung volumes receiving doses of ≥5 and ≥20 Gy, respectively, and the mean lung dose for PBT were 27.8%, 21.7%, and 11.1 Gy (RBE), respectively. PBT resulted in lower lung doses compared with x-rays in all cases, without any increase in the heart doses. PBT resulted in reductions of the median lung V5 and V20 and mean lung dose of 16.1%, 2.7%, and 1.9 Gy (RBE), respectively, compared with x-ray radiation therapy.

**Table 3:** Comparison of the lung and heart doses of radiation therapy using x-rays versus proton beam therapy.

Case	Primary tumor location	PTV (cm^3^)	Prescribed dose (Gy (RBE))	Lung V5 (%)		Lung V20 (%)		MLD (Gy (RBE))		Heart V5 (%)	
				x-ray	PBT	x-ray	PBT	x-ray	PBT	x-ray	PBT
1	Central	50.3	70.0	38.5	27.4	24.2	21.8	11.4	10.6	65.0	11.3
2	Central	20.8	72.6	41.0	31.9	24.6	24.3	11.8	11.6	4.3	0
3	Central	103.4	72.6	57.2	28.2	34.0	21.6	17.7	12.6	31.2	6.3
4	Peripheral	32.0	66.0	37.5	16.5	12.8	9.8	8.4	5.4	6.4	0

**Abbreviations:** MLD, mean lung dose; PBT, proton beam therapy; PTV, planning target volume; RBE, relative biological effectiveness; V5, percentage of the volume receiving ≥5 Gy; V20, percentage of the volume receiving ≥20 Gy.

## Discussion

Since treatment after pneumonectomy is risky, it is important to differentiate between a metachronous primary tumor and metastasis; however, this distinction is often challenging. Criteria for this determination include those proposed by Martini et al^1^ and Colice et al.^11^ In both criteria, key factors include whether the histology differs between the surgical lesion and recurrent lesion, the interval between treatment and development of the next tumor, and the presence of extrapulmonary metastases. Although 3 of the 4 cases had the same SCC histology in this study, more than 8 years had passed since pneumonectomy; thus, we classified these cases as metachronous primary tumors and treated them with definitive PBT with curative intent. Three of the 4 patients survived ≥3 years. This is supportive of the use of definitive radiation therapy doses in this setting.

Some reports have shown the effectiveness of radiation therapy after pneumonectomy. In a database study of 191 inoperable patients with metachronous or recurrent tumors in the contralateral lung after pneumonectomy, Ayub et al^6^ found that patients treated with radiation therapy had better survival compared with patients who did not receive radiation therapy, regardless of tumor size. Senthi et al^12^ reported a median survival of 39 months in 27 cases (15 T1N0M0, 8 T2N0M0, 3 T3N0M0, and 1 T2N1M0, according to the American Joint Commission on Cancer 6th classification) treated with radiation therapy for a second primary tumor after pneumonectomy. Likewise, definitive PBT may have contributed to the prolonged survival in the present 4 cases.

Radiation pneumonitis (RP) is a major concern when treating patients with definitive radiation therapy to the residual lung after pneumonectomy. In a systematic review evaluating the safety of SBRT after pneumonectomy, Arifin et al^7^ reported that the incidence of grade ≥3 AEs (eg, RP) was 13.2%. Thompson et al^13^ also reported that grade 3 RP occurred in 15% of 13 patients with SBRT after pneumonectomy. Because the lung volume is small after pneumonectomy, lung V5, V20, and mean lung dose, which are widely recognized factors associated with symptomatic RP, may need to be reduced more strictly. Testolin et al^14^ retrospectively analyzed 12 patients treated with SBRT (26-40 Gy in 1-4 fractions) for secondary lung tumors after pneumonectomy and reviewed previous reports; they suggested that by keeping the lung V5 < 50%, V20 < 7%, and mean lung dose <8 Gy, the risk of severe lung toxicity was acceptable. In addition, it may be required to reduce the dose to the mediastinum depending on tumor location. Senthi et al^12^ reported a higher incidence of grade ≥3 RP after SBRT following pneumonectomy when the lung dose was increased due to excessive concern about the dose to the mediastinum, indicating a trade-off between the lung and mediastinum doses. Although the target volume and prescribed doses in the 4 patients in our study were higher than those of previous reports of SBRT, and the lung doses in our cases did not meet the suggested dose constraints by Testolin et al,^14^ no grade ≥3 pneumonitis occurred. If the patients in this study had been treated with x-rays, the lung doses would have been even higher, potentially increasing the risk of severe lung toxicity. The data on radiation therapy to the contralateral lung after pneumonectomy are still limited, and further studies are needed to establish appropriate dose limitations.

In recent years, the impact of immune check point inhibitor therapy has been significant, and the number of pneumonectomies performed as salvage surgery or following conversion to resectability is likely to increase in coming years.^15^ In fact, pneumonectomy was performed in 11.8% of all patients in the KEYNOTE671 trial, in which preoperative chemotherapy plus immune check point inhibitors were used.^15^, ^16^ In addition, because of the cardiopulmonary burden of chemotherapy after pneumonectomy, there is a potential need for radiation therapy in cases of localized disease for which local treatment is considered worthwhile.^17^

## Conclusions

Definitive PBT for new lesions in the residual lung after pneumonectomy was successful without serious AEs. However, the number of cases was small, and more cases are needed.

## Ethics

All procedures performed in studies involving human participants were in accordance with the ethical standards of the institutional and/or national research committee and with the 1964 Helsinki Declaration and its later amendments or comparable ethical standards. This retrospective study was approved by the institutional review board of the University of Tsukuba Hospital (approval no. R06-118).

## Consent for publication

Written informed consent was obtained from the patients for publication.

## Funding

This work was supported by the University of Tsukuba.

## Author Contributions

Masatoshi Nakamura and Kayoko Ohnishi: Conceptualization, Methodology. Den Fujioka, Keiichiro Baba, and Motohiro Murakami: Investigation. Masatoshi Nakamura, Kayoko Ohnishi, Toshiyuki Okumura, Masashi Mizumoto, Yukio Sato, Ikuo Sekine, and Masaharu Inagaki: Resources. Masatoshi Nakamura, Kayoko Ohnishi, and Den Fujioka: Data curation. Masatoshi Nakamura: Writing- Original draft. Kayoko Ohnishi: Writing- Review and Editing. Hideyuki Sakurai: Supervision. All authors read and approved the final manuscript.

## Declaration of Conflicts of Interest

The authors declare that they have no known competing financial interests or personal relationships that could have appeared to influence the work reported in this paper.

## Data Availability

The data sets used and/or analyzed during the current study are available from the corresponding author on reasonable request.
